# Efficacy of tooth brushing via a three-dimensional motion tracking system for dental plaque control in school children: a randomized controlled clinical trial

**DOI:** 10.1186/s12903-022-02665-6

**Published:** 2022-12-22

**Authors:** Jin-Sun Jeong, Kyeong-Seop Kim, Jeong-Whan Lee, Kee-Deog Kim, Wonse Park

**Affiliations:** 1 School of Nursing and Rehabilitation, Cheeloo College of Medicine, Shandong University, Jinan, 250012 China; 2 Department of Biomedical Engineering, College of Science and Technology, Konkuk University, Chungju, Korea; 3 Department of Advanced General Dentistry, College of Dentistry, Yonsei University, 50-1 Yonsei-ro, Seodaemun-gu, Seoul, 03722 Korea

**Keywords:** Schoolchildren, Oral hygiene, Toothbrushing instruction, Computer, Smart toothbrush, Dental plaque

## Abstract

**Background:**

School children are in a developmental period in which permanent teeth replace primary dentition. It is also a period with a high incidence of gingivitis and caries, which can be improved with adequate tooth brushing. Advances in information technology have led to the development of smart health devices that assist in tooth brushing. We compared the effectiveness of computer-assisted toothbrushing using a toothbrushing instruction (TBI) method called the smart toothbrush and smart mirror (STM) system with that of conventional TBI (verbal instructions) for plaque control in school children.

**Methods:**

This randomized controlled clinical trial analyzed and compared the reduction of the modified Quigley-Hein plaque index between the two methods in 42 school children. The participants were randomly assigned to the STM system group (n = 21) or conventional-TBI group (n = 21). The plaque indices were evaluated at baseline, immediately after TBI (day 0), and 1 week and 1 month after TBI.

**Results:**

The STM system and conventional TBI led to an average reduction of 40.50% and 40.57%, respectively, in whole mouth plaque. Reductions in the plaque indices within each tested time period were observed in both groups (*P* < 0.001), and the mean plaque reduction did not differ between the two groups (*P* = 0.44).

**Conclusions:**

The present study tested a computer assisted system for TBI, more studies are needed to confirm its usefulness in different objectives.

*Clinical relevance* The computer-assisted STM system may be an alternative of TBI for children.

*Trial registration* ClinicalTrials.gov (NCT04627324) Registered 13/11/2020—Retrospectively registered, https://clinicaltrials.gov/ct2/show/NCT04627324.

## Introduction

According to the World Health Organization, oral diseases have a lifelong impact due to pain, discomfort, disfigurement, or even death in some cases [1]. Dental plaque is a biofilm or mass of bacteria that grows on intraoral surfaces. Cariogenic diet and failure to control dental plaque results in dental caries and periodontal diseases [2, 3], which are common worldwide [4–7]. Tooth brushing is a safe and efficacious technique for removal and prevention of dental plaque [8, 9].

During school-age years, new permanent teeth erupt, which are very rugged with deep pits [10, 11]. Given their shape, the teeth are vulnerable to caries. Tooth brushing during this time is difficult due to the presence of shaky and newly erupting teeth, both of which increase the risk for gingivitis [12, 13].

Parents’ management of their child’s tooth brushing and the child’s cooperation in the process are particularly important during this period [14–17]. However, parents may not be aware of the adequate tooth brushing technique and may not be able to visit the dentist to learn about this technique. Furthermore, even if they do know the correct technique, it is difficult for parents to supervise their child every time they brush their teeth [18]. In a study of Luciane et al., the importance of regular instruction for children was emphasized [19].

In recent years, advances in information technology (IT) have motivated the development of many smart health devices that enable more effective health management. Studies on the development of three-dimensional (3D) artificial joints and IT-based disease monitoring in patients with stroke and heart failure are underway [20–22]. In dentistry, electronic sonic toothbrushes and oscillating/pulsating toothbrushes, as well as devices that assist in tooth brushing, such as those that record tooth brushing time, have been developed [23–26]. Nevertheless, these devices do not evaluate whether the users are brushing their teeth properly and do not teach the users how to brush their teeth. The smart toothbrush and smart mirror (STM) system (XiuSolution, Gyeonggi-do, Korea) is one such smart health device that contains a 3D accelerometer and a magnetic sensor, and gives feedback on positioning and efficiency during toothbrushing [27–30].

The aim of this research was to compare the efficacy of a computer-assisted tooth brushing instruction (TBI) method, the STM system, with that of conventional TBI for plaque control in school children. Our hypothesis was that the efficacy of the STM system would be comparable with that of conventional TBI.

## Materials and methods

### Trial design

This was a parallel randomized control trial in which participants were assigned to either TBI with the STM system or the control group received conventional TBI (i.e., verbal instructions). The tooth brushing effect was evaluated at baseline, immediately after TBI (day 0), and 1 week and 1 month after TBI with the plaque indices. The trial was organized in accordance with the Consolidated Standards of Reporting Trials (CONSORT) guidelines.

### Participants

This study was part of a larger study that also included adult participants, the data for which have been published [27]. Both studies were approved by the institutional review board of the Yonsei University dental hospital on the same protocol (IRB Number: 2-2008-0005). This study was registered at ClinicalTrials.gov (NCT 04627324). The researcher posted recruitment posters at the Yonsei University Dental Hospital, and two public health centers in Gangseo-gu and Mapo-gu. In the poster, the following contents were included: the purpose of the study, number of visits, the required time for every visit, the inclusion (including age: 6–12 years) and the exclusion criteria, place of experiment, contact number. The participants were recruited in one of the following three institutes: the Yonsei University Dental Hospital (n = 15) and two public health centers in Gangseo-gu and Mapo-gu (n = 40). All the three institutes were located close to the participants’ residence; i.e., a 20-min walk from the residence to the institutes. The participants and their parents or guardians were provided verbal and written information about the products and purpose of the study, and written consent was obtained from the parents or guardians of the children during the first visit.

Participants were required to have at least one of the following symptoms of gingivitis, swollen gums, bright red or purple gums, gums that are tender or painful to the touch, gums that bleed easily when you brush or floss. And a baseline plaque scores > 1.5 (the Turesky Modification of the Quigley-Hein index) [31, 32] was also required. Participants were excluded if they had rampant caries [33] or orthodontic appliances, or were immunocompromised (such as systemic diseases, any adverse medical history, or long-term medication use).

### Tooth brushing Instruction System

**Fig. 1:**
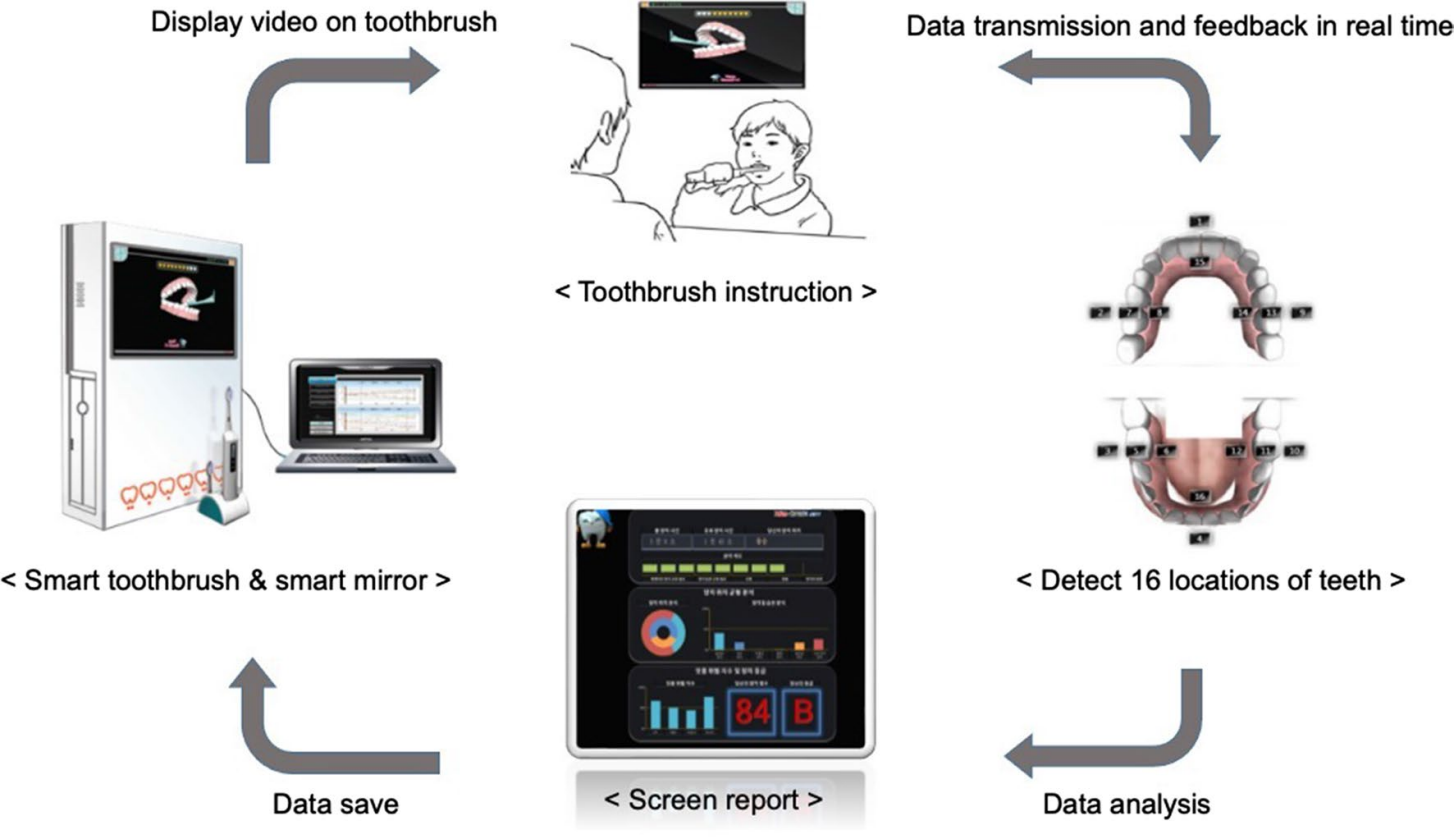
Schematic representation of smart toothbrush and smart mirror (STM) system

The STM system comprises a smart mirror, which is integrated into a computer monitor, and a 3D motion-capture device inside of a modified toothbrush holder. The computer program displays a toothbrushing animation in the mirror. Toothbrush motions detected by sensors embedded inside the holder are captured and sent to the server for analysis (Fig. 1). The toothbrush motions are seen on this website. The video at the following link demonstrates the toothbrush motions: https://www.youtube.com/watch?v=Cvoy4deM-VQ.

The STM system determines the tooth brushing pattern and provides real-time voice feedback such as “Good,” “Very Good,” and “Oops.” The level of TBI can be adjusted according to the user’s tooth brushing ability. Brushing areas are divided into 16 zones (anterior facial and lingual [palatal], right and left posterior occlusal, buccal, and lingual [palatal] for maxillary and mandibular teeth) (Fig. 1), and the duration of toothbrushing for each area can be set. The user can also select the method of brushing, from rolling, modified bass, or circle (Fones method). The color of the tooth in the mirror gradually changes to white according to the toothbrushing compliance and suitability. On completion, the STM system shows a summary report. In this study, we opted to use the rolling method as recommended by the Korean Dental Association, and the brushing duration of each area was set to 10 s.

### Interventions

Participants were assigned to either TBI with the STM system or the control group received conventional TBI. Both were provided with the same fluoride-containing toothpaste (2080, Aekyung, Korea) and toothbrushes with a flat-trimmed nylon bristle of identical type and length. Both TBIs were formulated by one dental hygienist. Participants had to brush their teeth at home or school for three minutes, three times daily, and after meals, as recommended by the Korean Dental Association.

### Outcomes

The primary outcome was an at least 15% reduction in terms of Q-H plaque index in teeth cleaning effective before and after education, which could be regard as a significant plaque reduction by the American Dental Association (ADA) guideline [34]. Secondary outcome was statistical changes between two groups regarding Q-H plaque index, in terms of whole mouth, buccal and lingual surface, the buccal surface of the maxillary first molars, respectively.

### Plaque scoring

Participants first swished with a disclosing solution (2-Tone Disclosing Agent, Young Dental) for one minute. The Turesky Modification of the Quigley-Hein index was assessed by visual inspection after mouth gargling and confirmed with photography. Plaque examination was scored on the buccal and lingual surface of all the teeth (if a permanent tooth had not erupted yet, the primary tooth in the corresponding area was tested). The Turesky Modification of the Quigley-Hein index was scored between 0 and 5 (0: no plaque; 1: separate flecks of plaque at the cervical margin of the tooth; 2: a thin continuous band of plaque at the cervical margin of the tooth ≤ 1 mm; 3: plaque covering up to one-third of the tooth crown; 4: plaque covering between one-third and two-thirds of the tooth crown; and 5: plaque covering ≥ two-thirds of the tooth crown).

The percentage reduction in plaque scores for the buccal surface, lingual surface, and whole mouth was determined as follows:

$$\begin{aligned} & {\text{Percentage reduction in plaque score}} \\ & \quad = \frac{{{\text{prebrushing}}\left( {{\text{baseline}}} \right){\text{ plaque score }} - {\text{ postbrushing plaque score}}}}{{{\text{prebrushing}}\left( {{\text{baseline}}} \right){\text{plaque score}}}} \\ \end{aligned}$$

The examiner was trained and calibrated, exhibiting an intra-rater reliability kappa of 0.81 for the plaque index (Landis and Koch kappa-test, *p* < 0.001).

### Data collection

Participants visited the institutes three times. During the initial visit, participants underwent plaque examination and were then instructed using the STM system (used only during the appointment) or conventional TBI, according to their assigned group, followed by a re-evaluation of the plaque index. Subsequent visits were made at approximately 1 week (6–8 days) and 1 month (28–32 days) after the first visit to examine the maintenance and effectiveness of the TBI. All participants brushed their teeth at home and did not eat or drink before the plaque index was checked. Forty-two participants completed every visit.

### Sample size

Sample size calculation was based on the mean values and standard deviations [SD] of overall plaque scores provided by Kim et al., 2015 [27]. A sample size of 21 participants was required per group to ensure a power of 90% (with two-sided α = 0.05) or a greater chance of detecting differences of 3.07% plaque reduction between the two groups, assuming 15.94% SD and 10% dropouts after randomization.

### Randomization and blinding

Participants were randomly assigned into two equal-sized groups by flipping a coin. The experimental group received TBI with the STM system, while the control group received conventional TBI (i.e., verbal instructions). Another dental hygienist who was blinded to the group assignment determined the plaque status twice during the first visit and once during each subsequent visit.

### Statistical methods

Statistical analyses were performed using IBM SPSS (version 25, IBM Corp). The balance evaluation for the metric variables were analyzed by Kolmogorov–Smirnov test. Linear mixed model (LMM) was used for repeated measure the data that fit the balance, and Mann–Whitney U test was performed for data that did not fit the balance, and all data were assessed the change over time (baseline, after instruction [day 0], and 1 week and 1 month later).

A 15% statistically significant plaque reduction is needed to provide evidence of greater effectiveness in teeth cleaning [34]. Data are presented as mean (SD) unless otherwise indicated, and the alpha level was set at *p* < 0.05.

## Results

**Fig. 2:**
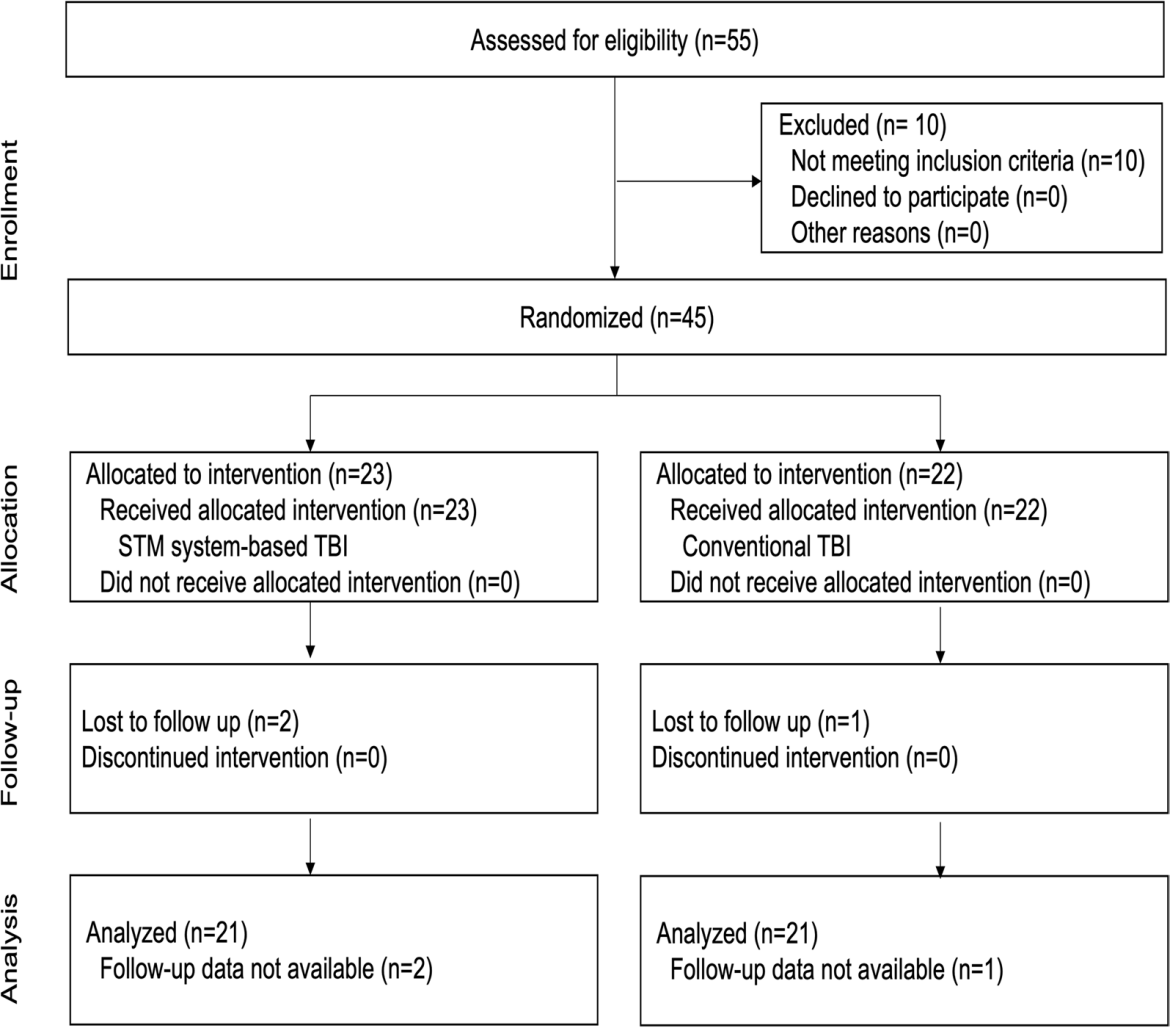
Study CONSORT diagram

The flow of participants is shown in Fig. 2. Of the 55 Korean children who were assessed, 10 participants did not meet the baselines scores. Therefore, 45 participants were finally recruited in the study, of which 42 completed the protocol and three dropped out for reasons unrelated to the study. Forty-two participants (female 21, male 21; mean age and SD (9.64 and 1.96 years), [range, 6–12 years]) completed the study. The minimum and maximum number of teeth were 12 and 28, respectively (mean 21.93, SD 4.06). Most participants were right-handed (93%) (Table 1).

**Table 1:** Patient demographics (N = 42)

Characteristics	Total	STM system TBI	Conventional TBI
*Age (years)*			
Mean (SD)	9.64 (1.96)	9.57 (1.87)	9.71 (2.00)
Range	6–12	6–12	6–12
*Sex*			
Female	21 (50%)	10 (48%)	11 (52%)
Male	21 (50%)	11 (52%)	10 (48%)
*Hand typically used for brushing*			
Right	39 (93%)	20 (95%)	19 (90%)
Left	3 (7%)	1 (5%)	2 (10%)
Ambidextrous	0 (0%)	0 (0%)	0 (0%)
*Number of teeth*			
Mean (SD)	21.93 (4.06)	21.52 (4.15)	22.33 (3.83)
Range	12(5)^a^–28(28)^a^	13(6)^a^–28(28)^a^	12(5)^a^–28(28)^a^

Data are presented as N (%) unless otherwise stated

*STM system* smart toothbrush and smart mirror system, *TBI* toothbrushing instruction, *SD* standard deviation

^a^Number of permanent teeth

### Primary outcome variable

The mean whole mouth prebrushing plaque scores for the STM system and conventional TBI groups were 3.31 and 3.21, respectively, and all participants exhibited gingivitis with different status. Whole mouth changes in the plaque index are presented in Table 2.

**Table 2:** Plaque indices and plaque reduction percentages for the whole mouth

	STM system TBI	Conventional TBI
Baseline	3.31 (0.80)	3.21 (0.81)
Immediately after TBI	1.99 (0.73)	2.06 (0.90)
1 week after TBI	2.01 (0.78)	2.12 (0.98)
1 month after TBI	1.88 (0.67)	1.72 (0.95)
Mean decrease	40.50%	40.57%
*p* value^a^	0.44	

*STM system* smart toothbrush and smart mirror system, *TBI* toothbrushing instruction

^a^Linear mixed model for repeated measure data, group x time interaction

A linear mixed model revealed a significant effect of time (*p* < 0.001). The whole mouth plaque index decreased from 3.31 at baseline to 1.88 one month after TBI for the STM system group, and from 3.21 to 1.72 for the same time period for the conventional TBI group. The mean percentage plaque score reductions for the STM system group and conventional TBI group over the entire study period were 40.5% and 40.57%, respectively. The mean percentage plaque score reductions for the STM system group and the conventional TBI group were 46.69% and 43.83%, respectively, for the buccal surfaces, and 31.05% and 33.89%, respectively, for the lingual surfaces (Table 3).

**Table 3:** Plaque indices and plaque reduction percentages for the buccal and lingual surfaces

	Buccal		Lingual	
	STM system TBI	Conventional TBI	STM system TBI	Conventional TBI
Baseline	3.77 (0.92)	3.65 (0.88)	2.86 (0.90)	2.76 (0.96)
Immediately after TBI	2.03 (0.78)	2.12 (0.99)	1.96 (0.79)	1.99 (0.97)
1 week after TBI	2.11 (0.97)	2.24 (1.07)	1.91 (0.81)	2.01 (0.96)
1 month after TBI	1.91 (0.90)	1.90 (1.13)	1.86 (0.65)	1.54 (0.82)
Mean decrease	46.69%	43.83%	31.05%	33.89%
*p* value^a^	0.78		0.25	

*STM system* smart toothbrush and smart mirror system, *TBI* toothbrushing instruction

^a^Linear mixed model for repeated measure data, group x time interaction

### Secondary outcome variables

No effect of group (*p* = 0.44) (time X group) with respect to whole mouth plaque indices. There were no statistically significant differences in the effects of plaque removal on either the buccal or lingual sides between the two groups (*p* = 0.78 and *p* = 0.25, respectively).

A sub-analysis of the buccal surface of the maxillary first molars revealed that although the STM system group tended to show a greater plaque index reduction than the conventional TBI group immediately and 1 month after TBI, these differences failed to reach statistical significance (Table. 4).

**Table 4:** Plaque indices and plaque reduction percentages for buccal surface of the maxillary first molars

	STM system TBI	Conventional TBI	*p* value^a^
Baseline	4.29 (0.78)	4.10 (1.09)	0.725
Immediately after TBI	2.19 (1.04)	2.71 (1.33)	0.142
1 week after TBI	3.19 (1.09)	3.12 (1.31)	0.970
1 month after TBI	2.76 (1.26)	3.07 (1.43)	0.432
Mean decrease	37.35%	30.07%	

*STM system* smart toothbrush and smart mirror system, *TBI* toothbrushing instruction

^a^Mann-Whitney U test

### Adverse events

No participant reported any equipment-related physical harm.

## Discussion

### Principal results

The effects of computer-assisted TBI and conventional TBI on dental plaque in school children were evaluated in this study. The STM system and conventional TBI led to an average reduction of 40.50% and 40.57%, respectively, in whole mouth plaque. Both were, therefore, effective for teaching children how to brush their teeth. While the plaque index differed significantly across time points for both systems (*p* < 0.001), there was no significant difference in the plaque index between the two groups (*p* = 0.44).

Both groups showed greater plaque improvement in the buccal than in the lingual region. However, the greater effect on the lingual side in the control group at 1 month was possibly influenced by the Pygmalion effect. We surmise that the participants who had not brushed well on the lingual side at 1 week paid more attention to brushing this side in the ensuing weeks in anticipation of the 1-month evaluation.

For the buccal surface of the maxillary first molars, which is an area often neglected during tooth brushing by children [35], the STM system was actually more effective than the conventional TBI. This suggests that the system increases the effectiveness of tooth brushing on surfaces that are difficult to clean.

### Comparison with previous work

Manual toothbrushes are useful for the mechanical removal of dental plaque, which is known to be the primary cause of caries and periodontal disease [36]. However, effective toothbrushing is a learned skill that requires practice and professional TBI. Previous studies regarding the dissemination of TBI via mass media programs, posters, plays, or videos reported unsatisfactory results due to insufficient repetition and reinforcement [37–39].

In this study, the STM system, which is capable of detecting 3D motion (motion measured at each tooth position and at each area being brushed with real-time feedback and correction of toothbrushing technique), was examined as an alternative method for TBI that treats brushing like a game. In fact, while children are commonly not good at brushing, when they learn it as if it were a game, they actually become proficient at it [40, 41].

Although similar systems have been tested in several other studies, the experimental procedures were performed in either a clinical environment or examined adults [23, 27, 29]. Toothbrushing habits are established early in childhood, and children tend to have high levels of plaque, indicating that this is a particularly important period to target with effective TBI [42]. This was the motivation for using children in this study.

Our study results are consistent with those of Kim et al. [27] who found a 39.88% reduction in the plaque index using a similar system. Other methods have included leaflet-based and videotape-based TBI, which showed plaque reduction rates of 58% and 37%, respectively [43]. The discrepancy in plaque reduction rates in different studies may be due to differences in the baseline plaque index between the participants or differences in the plaque index systems used to evaluate oral cleanliness.

There are several recent studies on the use of power toothbrushes for dental plaque removal [44, 45]. Both power and manual toothbrushes are effective in plaque removal [46, 47]. The STM system is a manual toothbrush connected to a computerized handle for dental plaque removal. The computerized handle does not vibrate automatically, but only detects movement and gives feedback on positioning and efficiency during toothbrushing. The STM system can clean all parts of the teeth evenly in an appropriate amount of time. Additionally, it allows users to select the method of tooth brushing (modified bass, rolling, and the circle method) according to their oral health. This system is designed as a fun and easy tool to teach school children how to brush their teeth in lieu of busy medical staff in dental hospitals or parents at home.

Diverse studies utilizing telemedicine have recently been published. Particularly, the advances in mobile devices have led to more active research on the use of mobile devices for patients with cancer and heart disease [48, 49]. The manufacturer of the STM system has developed a mobile application, and it would be advisable to conduct further studies based on this mobile application.

### Limitations

The reported positive effects of the computer-assisted TBI on the improvement of tooth brushing skills and dental plaque control must be considered in light of several limitations. First, this was a short-term study, and the participants used the STM system only during the test visits. More accurate results would have been obtained if the participants had continued to use the system at home as well. Second, further studies may be required to assess the correlation between the summary scores and the degree of dental plaque control achieved. Lastly, the present study failed to collect the data on SES and patent education, as well as on caries rate/DMFS. The future study should mend the limitations above mentioned.

## Conclusion

TBI is highly important for maintaining oral hygiene and preventing caries and other dental diseases in children. The STM system exhibited efficacy in plaque control and may be a potential alternative for conventional TBI.

## Data Availability

The data that support the findings of this study are available from the corresponding author upon reasonable request.

## Abbreviations

IT: Information technology
STM system: Smart toothbrush and smart mirror system
TBI: Toothbrushing instruction
3D: Three-dimensional
